# Kazakh adults in Xinjiang have a prevalent obesity problem but a low prevalence of diabetes

**DOI:** 10.1186/s12889-024-18228-z

**Published:** 2024-03-04

**Authors:** Ruiting Shen, Sheng Jiang, Ruirui Cheng, Jinhui Zhuge, Xiaoxiao Li, Hua Yao, Mingchen Zhang

**Affiliations:** 1 Department of Endocrinology and Metabolism, Ningbo No.2 Hospital, Ningbo, Zhejiang Province China; 2 Department of Endocrinology, The First Affiliated Hospital of Xinjiang Medical University, Urumqi, China; 3 College of Public Health, Xinjiang Medical University, Urumqi, China; 4 Center of Health Management, The First Affiliated Hospital, Xinjiang Medical University, Urumqi, China

**Keywords:** Prevalence, Diabetes, Obesity

## Abstract

**Background:**

The prevalence of diabetes and obesity has been continuously rising worldwide over the last three decades, particularly in China. The prevalence varies widely among different ethnicities. In this study, we investigated the prevalence of diabetes and obesity, as well as the associated factors for diabetes in Kazakh adults in Xinjiang to improve diabetes screening.

**Methods:**

We collected data from the Xinjiang physical examination in 2018, including a total sample of 118,505 Kazakh adults in Altay District. Data on demographic characteristics, medical history, physical examination, fasting plasma glucose (FPG) and serum lipid profiles were collected. The chi-square test was used to examine the differences between multiple variables. Multivariate logistic regression was performed to identify the factors associated with diabetes.

**Results:**

The mean age was 43. 66 years (SD 14.14). 49.3% of the population were women and 75.5% were rural residents. The mean FPG was 5.33 mmol/L (SD 1.22). The prevalence of diabetes was 6.3% and 4.1% received a new diagnosis by FPG. 26.6% were diagnosed with impaired fasting glucose (IFG). The mean body mass index (BMI) was 26.29 kg/m^2^ (SD 14.14) and the mean waist circumference was 87.69 cm (SD 12.74). 33.2% of the population were overweight, and 33.0% were obese. The prevalence of central obesity was 51.4%. Diabetes was mostly positively associated with hypertension (OR = 3.821, *P*<0.001), hypertriglyceridemia (OR = 2.757, *P*<0.001), and hyper-LDL-cholesterolemia (OR = 2.331, *P*<0.001) in the Kazakh population. The ORs for overweight, obesity and central obesity predictive of diabetes were 1.265, 1.453 and 1.222 ( all *P*<0.001), respectively.

**Conclusions:**

Despite having a high prevalence of obesity and central obesity, the Kazakh population had a considerably low prevalence of diabetes. Obesity was not the most important risk factor for diabetes in Kazakh individuals. The awareness of diabetes was low. When screening for diabetes in Kazakhs, those with hypertension or dyslipidemia should receive more attention.

**Supplementary Information:**

The online version contains supplementary material available at 10.1186/s12889-024-18228-z.

## Introduction

The prevalence of diabetes and obesity worldwide has been continuously rising in the last three decades. With 10.5% of the world’s adult population now having diabetes and 5.8% having impaired fasting glucose (IFG), the global burden of the disease has substantially expanded [1, 2]. The worldwide obesity prevalence was 13% in adults and 39% in overweight individuals in 2016 [3]. The largest proportion of global metabolic-related mortality and disability-adjusted life years was due to obesity [4]. The prevalence of diabetes in China increased from less than 1% in the 1980s to 12.4% in 2018 [5]. The most recent nationwide study in China shows that 34.8% of the population were overweight and 14.1% were obese in 2019 [6]. The prevalences are different in various ethnicities. A previous study of 3919 Kazakhs and 5583 Hans in Xinjiang China from 2007 to 2010 showed that 3.6% of Kazakh adults and 9.3% of Han adults had type 2 diabetes. Meanwhile, Kazakh individuals had a higher body mass index (BMI) and waist circumference compared to Han individuals [7]. Altay is one of the largest inhabited areas of Kazakh ethnicity in Xinjiang, China. A total of 52.76% of the Altay population is Kazakh. This study was conducted to survey the recent prevalence of diabetes and obesity in the Kazakh adult population in the Altay District of Xinjiang.

## Methods

### Subjects and sampling

The Xinjiang physical examination is a free physical examination provided by the Chinese government to all Xinjiang residents. A total of 201,438 Kazakhs participated in the physical examination of Altay in 2018 were recruited. Kazakh subjects who met the following inclusion criteria were eligible to participate in the study: (1) aged 18 and above; (2) living in Altay for at least 6 months. This study was approved by the Ethics Committee of the First Affiliated Hospital of Xinjiang Medical University (20160316-02). Individual informed consent was waived, as only anonymized data were used in this study, which was approved by the Ethics Committee of the First Affiliated Hospital of Xinjiang Medical University. After data cleaning and checking, 118,505 were eventually included. Among the excluded participants, 25,158 did not complete the questionnaire. 10,709 did not complete the physical examination. 47,066 lacked blood indexes.

### Data collection

All investigators were community medical staff and underwent unified training prior to the start of the study. Data collection included face-to-face questionnaire interviews, clinical physical examinations and venous blood collection. To collect data on demographic characteristics (age, gender, ethnic) and medical history (history of diabetes, hypertension and dyslipidemia), a standard questionnaire was used. The ethnic of the participants was self-reported. Body weight, height, waist circumference, and blood pressure were measured in physical examinations. An electronic blood pressure monitor was used to measure blood pressure on the non-dominant arm twice in a row, with a 10 min interval, and the participant was seated after five minutes of rest. BMI was calculated as weight divided by height squared (kg/m^2^). Blood samples were collected after an overnight fast of at least eight hours. Fasting plasma glucose (FPG) and serum lipid profiles, including triglycerides (TG), total cholesterol (TC), low-density lipoprotein (LDL), and high-density lipoprotein (HDL) were measured in the medical examinations.

### Diagnostic criteria

Compared to the international criteria, lower BMI cut points are used in some Asian nations to define overweight and obesity as the relationship between BMI and comorbidities differs by population and ethnicity. According to the Chinese criteria, normal weight was defined as BMI < 24 kg/m^2^, while obesity was defined as BMI ≥ 28 kg/m^2^. Individuals with a BMI ≥ 24 kg/m^2^ and < 28 kg/m^2^ were classified as overweight [8]. Central obesity was defined as waist circumference ≥ 85 cm and ≥ 90 cm for women and men, respectively. Hypertension was defined as systolic blood pressure (SBP) ≥ 140 mmHg, diastolic blood pressure (DBP) ≥ 90 mmHg, or a self-reported previous diagnosis of hypertension. Residents without a prior diagnosis of diabetes were divided into normal fasting glucose, (IFG, and diabetes mellitus. Residents with FPG < 5.6 mmol/L (100 mg/dL) were identified as having normal fasting glucose. FPG levels of 5.6 to 6.9 mmol/L (100 to 125 mg/dL) identified residents with IFG. Residents with FPG ≥ 7.0 mmol/L (126 mg/dL) were identified as having diabetes. Diagnosed diabetes was recognized as a self-reported diagnosis that had already been made by a medical professional. Hypertriglyceridemia was defined as TG ≥ 2.3 mmol/L. Hypercholesterolemia was defined as TC ≥ 6.2 mmol/L. Hyper-LDL-cholesterolemia was defined as LDL ≥ 4.1 mmol/L and hypo-HDL- cholesterolemia was defined as HDL < 1.0 mmol/L. Dyslipidemia was defined as TG ≥ 2.3 mmol/L, TC ≥ 6.2 mmol/L, LDL ≥ 4.1 mmol/L, HDL < 1.0 mmol/L or self-reported use of lipid-lowering medications in accordance with the 2016 Chinese Adult Dyslipidemia Prevention Guideline [9].

### Statistical analysis

SPSS software (version 21.0) was used for the statistical analyses. *P* value < 0.05 indicated significance. Continuous variables are expressed as the mean ± SD. Student’s t test or the Mann-Whitney U test was used for continuous variables normally distributed or not, respectively. Categorical variables are presented as percentages (%) and were analyzed using the Chi-square test. A multivariate logistic-regression analysis was used to determine the adjusted odds ratios (ORs) and 95% CIs of the independent predictors of diabetes and IFG.

## Results

### Characteristics of the study subjects

Among the 118,505 surveyed subjects, 49.3% were women, and 75.5% were rural residents. The mean age was 43. 66 years (SD 14.14). The mean FPG was 5.33 mmol/L (SD 1.22). The mean BMI was 26.29 kg/m^2^ (SD 14.14) and the mean waist circumference was 87.69 cm (SD 12.74). 35.0% of the population had hypertension and 27.3% had dyslipidemia (Table 1).

**Table 1:** Characteristics of the Kazakh Population in Altay

Characteristics	Overall	Normal Glucose	Total Diabetes*	Diagnosed Diabetes	New-diagnosed Diabetes Based on FPG	IFG
N (%)	118,505	79,582(67.2)	7,455(6.3)	2,572(2.2)	4,883(4.1)	31,468(26.6)
Age (years)	43.66 ± 14.14	42.11 ± 13.79	55.61 ± 13.41	60.23 ± 11.63	53.17 ± 13.64	44.76 ± 13.73
Male/female (%)	50.7/49.3	51.5/48.5	47.8/52.2	54.2/45.8	44.4/55.6	44.4/55.6
Urban/rural (%)	24.5/75.5	25.7/74.3	25.2/74.8	25.9/74.1	24.9/75.1	21.1/78.9
FPG (mmol/L)	5.33 ± 1.22	4.81 ± 0.51	8.14 ± 2.84	7.67 ± 3.70	8.39 ± 2.22	5.96 ± 0.31
BMI (kg/m2)	26.29 ± 4.44	25.86 ± 4.33	28.52 ± 4.63	28.87 ± 4.69	28.33 ± 4.58	26.85 ± 4.44
Waist circumference (cm)	87.69 ± 12.74	86.81 ± 12.26	93.84 ± 13.95	95.89 ± 13.40	92.77 ± 14.11	88.44 ± 13.16
Hypertension (%)	41,485(35.0)	24,814(31.2)	5,854(78.5)	2,109(82.0)	3,745(76.7)	10,817(34.4)
SBP (mmHg)	127.78 ± 21.23	125.82 ± 20.46	141.88 ± 23.53	142.52 ± 24.13	141.55 ± 23.20	129.40 ± 21.27
DBP (mmHg)	77.31 ± 13.03	76.39 ± 12.80	84.80 ± 13.78	83.72 ± 13.74	85.37 ± 13.77	77.87 ± 12.83
Dyslipidemia (%)	32,381(27.3)	18,993(23.9)	3,603(48.3)	1,113(43.3)	2,490(51.0)	9,845(31.3)
TC (mmol/L)	4.90 ± 1.18	4.80 ± 1.15	5.41 ± 1.29	5.24 ± 1.35	5.51 ± 1.25	5.05 ± 1.17
TG (mmol/L)	1.31 ± 1.06	1.22 ± 0.94	1.86 ± 1.55	1.73 ± 1.38	1.94 ± 1.63	1.41 ± 1.17
LDL (mmol/L)	2.35 ± 1.03	2.30 ± 0.98	2.91 ± 1.16	2.68 ± 1.16	3.03 ± 1.14	2.35 ± 1.05
HDL (mmol/L)	1.48 ± 0.42	1.48 ± 0.42	1.42 ± 0.41	1.45 ± 0.43	1.41 ± 0.40	1.48 ± 0.42

* Total diabetes includes diagnosed diabetes and new-diagnosed diabetes based on FPG. Abbreviation: FPG, fasting plasma glucose; IFG, impaired fasting glucose; BMI, body mass index; SBP, systolic blood pressure; DBP, diastolic blood pressure; TC, total cholesterol; TG, triglycerides; LDL, low-density lipoprotein; HDL, high-density lipoprotein

### Prevalence of diabetes and IFG

The overall prevalence of total diabetes in Kazakh adults was 6.3%, with 6.5% in men and 6.1% in women (Table 2). The age and sex standardized prevalence was 5.4%, with 5.8% in men and 5.0% in women. There were no significant differences between rural and urban residents. The prevalence of diabetes increased with BMI (*P* < 0.001) and was 3.1% in the normal group, 6.0% in the overweight group and 9.8% in the obesity group. In those with central obesity, the prevalence of diabetes was 8.5%, compared to 4.0% in people without central obesity (*P* < 0.001). The prevalence of diabetes in the hypertensive group (14.1%) was significantly higher than that in nonhypertensive group (2.1%) (*P* < 0.001). In the dyslipidemia group, the prevalence of diabetes was substantially greater (11.1%) than that in the non-dyslipidemia group (4.5%) (*P* < 0.001). 4.1% of the overall population, 4.5% of males, and 3.7% of females were newly diagnosed with diabetes. These individuals made up 65.5%, 69.8%, and 60.8% of the total diabetic population, respectively. It was higher in males than in females (*P* < 0.001). With aging comes an increase in newly diagnosed diabetes, especially after the age of 50.

The prevalence of IFG was 26.6% in the overall population, 29.2% in men and 23.9% in women. The standardized prevalence was 25.9%, with 28.4% in men and 23.2% in women. Compared by sex, the prevalence of IFG was higher in males (*P* < 0.05).

**Table 2:** Prevalence of Diabetes and IFG in Different Groups (n (%))

Group	Total Diabetes	Diagnosed Diabetes	New-diagnosed Diabetes Based on FPG	IFG
Overall	7,455 (6.3, 5.4^△^)	2,572 (2.2, 1.9^△^)	4,883 (4.1, 3.3^△^)	31,468 (26.6, 19.5^△^)
Sex				
Women	3,561(6.1)	1,395(2.4)	2,166(3.7)	13,959(23.9)
Men	3,894(6.5)	1,177(2.0)	2,717(4.5)	17,509(29.2)
Age(years)				
18–29	128(0.6)	2(0.0)	126(0.6)	4,354(21.8)
30–39	747(2.5)	85(0.3)	662(2.2)	7,711(25.4)
40–49	1,644(5.3)	389(1.3)	1,255(4.0)	8,693(28.2)
50–59	2,066(10.3)	764(3.8)	1,302(6.5)	5,803(29.0)
60–69	1,708(14.9)	779(6.8)	929(8.1)	3,329(29.0)
≥ 70	1,162(19.7)	553(9.4)	609(10.3)	1,578(26.8)
Location				
Rural	5,574(6.2)	1,905(2.1)	3,669(4.1)	24,826(27.7)
Urban	1,881(6.5)	667(2.3)	1,214(4.2)	6,642(7.4)
BMI (kg/m2)				
<24 (Normal)	1,262(3.1)	414(1.0)	848(2.1)	8,945(22.3)
24-<28 (Overweight)	2,373(6.0)	745(1.9)	1,628(4.1)	10,678(27.1)
≥ 28 (Obese)	3,820(9.8)	1,413(3.6)	2,407(6.2)	11,845(30.3)
Central obesity				
Yes	5,176(8.5)	1,963(3.2)	3,213(5.3)	16,828(27.6)
No	2,279(4.0)	609(1.1)	1,670(2.9)	14,640(25.4)
Hypertension				
Yes	5,854(14.1)	2,109(5.1)	3,745(9.0)	10,817(26.1)
No	1,601(2.1)	463(0.6)	1,138(1.5)	20,651(26.8)
Dyslipidemia				
Yes	3,603(11.1)	1,113(3.4)	2,490(7.7)	9,843(30.4)
No	3,852(4.5)	1,459(1.7)	2,393(2.8)	21,625(25.1)

^△^age and sex standardized prevalence. Crude prevalence is unlabeled. Abbreviation: FPG, fasting plasma glucose; IFG, impaired fasting glucose; BMI, body mass index

### Prevalence of obesity and central obesity

33.2% of the population were overweight, and 33.0% were obese. The prevalence of central obesity was 51.4%, with 49.6% in men and 53.2% in women (Table 3). The age and sex standardized prevalences of overweight, obesity and central obesity were 32.8%, 34.2% and 53.5%, respectively. The prevalence of obesity was 29.4% in the normal glucose group, 37.6% in the IFG group and 51.2% in the diabetes group (*P* < 0.001). Central obesity made up 69.4% of individuals with diabetes, and 48.9% of individuals with normal glucose (*P* < 0.001). The prevalence of obesity and central obesity in the hypertensive group (47.7% and 65.4%) was significantly higher than that in the non-hypertensive group (25.1% and 43.8%) (*P* < 0.001). In the dyslipidemia group, the prevalence of obesity was greater (42.7% and 60.1%) than that in the non-dyslipidemia group (29.3% and 48.1%) (*P* < 0.001).

**Table 3:** Prevalence of Obesity and Central Obesity in Different Groups (n (%))

Group	BMI (kg/m2)			Central obesity	
	<24 (Normal)	24-<28 (Overweight)	≥ 28 (Obese)	Yes	No
Overall	40,081(33.8)	39,332(33.2)	39,092(33.0)	60,881(51.4)	57,624(48.6)
Age(years)					
18–29	11,892(59.5)	5,390(27.0)	2,694(13.5)	5,706(28.6)	14,270(71.4)
30–39	11,357(37.4)	10,567(34.8)	8,416(27.7)	13,762(45.4)	16,578(54.6)
40–49	8,091(26.2)	11,032(35.8)	11,701(38.0)	17,447(56.6)	13,377(43.4)
50–59	4,387(21.9)	6,585(32.9)	9,034(45.2)	12,901(64.5)	7,105(35.5)
60–69	2,618(22.8)	3,774(32.9)	5,072(44.2)	7,496(65.4)	3,968(34.6)
≥ 70	1,736(29.4)	1,984(33.7)	2,175(36.9)	3,569(60.5)	2,326(39.5)
Sex					
Women	21,204(36.2)	18,281(31.3)	18,984(32.5)	31,089(53.2)	27,380(46.8)
Men	18,877(31.4)	21,051(35.1)	20,108(33.5)	29,792(49.6)	30,244(50.4)
Location					
Rural	30,618(34.2)	29,742(33.2)	29,149(32.6)	45,718(51.1)	43,791(48.9)
Urban	9,463(32.6)	9,590(33.1)	9,943(34.3)	15,163(52.3)	13,833(47.7)
Diabetes					
Total Diabetes	1,262(16.9)	2,373(31.8)	3,820(51.2)	5,176(69.4)	2,279(30.6)
Diagnosed Diabetes	414(16.1)	745(29.0)	1,413(54.9)	1,963(76.3)	609(23.7)
New-diagnosed Diabetes Based on FPG	848(17.4)	1,628(33.3)	2,407(49.3)	3,213(65.8)	1,670(34.2)
IFG	8,945(28.4)	10,678(33.9)	11,845(37.6)	16,828(53.5)	14,640(46.5)
Normal Glucose	29,874(37.6)	26,281(33.0)	23,427(29.4)	38,877(48.9)	40,705(51.1)
Hypertension					
Yes	8,344(20.1)	13,350(32.2)	19,791(47.7)	27,147(65.4)	14,338(34.6)
No	31,737(41.2)	25,982(33.7)	19,301(25.1)	33,734(43.8)	43,286(56.2)
Dyslipidemia					
Yes	7,762(24.0)	10,793(33.3)	13,826(42.7)	19,453(60.1)	12,928(39.9)
No	32,319(37.5)	28,539(33.1)	25,266(29.3)	41,428(48.1)	44,696(51.9)

Abbreviation: BMI, body mass index; FPG, fasting plasma glucose; IFG, impaired fasting glucose

### Risk factors of diabetes

Multivariate logistic-regression analysis revealed that diabetes was mostly positively associated with hypertension (OR = 3.821, *P*<0.001), hypertriglyceridemia (OR = 2.757, *P*<0.001), and hyper-LDL-cholesterolemia (OR = 2.331, *P*<0.001) in the Kazakh population (Table 4). The ORs of obesity, overweight and central obesity were 1.453 (*P*<0.001), 1.265 (*P*<0.001) and 1.222 (*P*<0.001), respectively. The ORs adjusted for age, sex and location were shown in Appendix Table 1. Multivariate logistic regression was performed in male and female groups respectively. Hypertension, hypertriglyceridemia and hyper-LDL-cholesterolemia were the most significant risk factors of diabetes in both groups (Appendix Table 2). The results of multivariate logistic regression in normal weight, overweight and obesity groups were shown in Appendix Table 3.

IFG was positively associated with obesity (OR = 1.556, *P*<0.001), rural residence (OR = 1.322, *P*<0.001) and overweight (OR = 1.283, *P*<0.001).

**Table 4:** Risk factors for total diabetes and IFG (odds ratio (95% confidence interval))

Variables	Diabetes		IFG	
	OR(95%CI)	*P*	OR(95%CI)	*P*
Females sex (vs. males)	0.944(0.897–0.993)	0.025	0.765(0.745–0.786)	<0.001
Age	1.039(1.036–1.041)	<0.001	1.010(1.009–1.012)	<0.001
Rural (vs. urban)	0.985(0.930–1.043)	0.604	1.322(1.282–1.364)	<0.001
Overweight	1.265(1.172–1.365)	<0.001	1.283(1.240–1.328)	<0.001
Obesity	1.453(1.344–1.570)	<0.001	1.556(1.496–1.617)	<0.001
Central obesity	1.222(1.150–1.299)	<0.001	0.911(0.883–0.940)	<0.001
Hypertension	3.821(3.585–4.073)	<0.001	0.742(0.718–0.766)	<0.001
Hypercholesterolemia	1.091(1.022–1.164)	0.009	1.212(1.165–1.261)	<0.001
Hypertriglyceridemia	2.757(2.581–2.945)	<0.001	1.172(1.120–1.227)	<0.001
Hyper-LDL-cholesterolemia	2.331(2.161–2.515)	<0.001	0.962(0.909–1.018)	0.183
Hypo-HDL cholesterolemia	1.216(1.111–1.330)	<0.001	1.110(1.058–1.166)	<0.001

Abbreviation: IFG, impaired fasting glucose; LDL, low-density lipoprotein; HDL, high-density lipoprotein

## Discussion

Our data from 118,505 Kazakh adults in Altay showed that the Kazakh population had a low prevalence of diabetes (6.3%) but a high BMI (26.29 kg/m^2^) and waist circumference (87.69 cm). According to the Chinese criteria, 33.2% of the population had overweight and 33.0% had obesity. Our results are generally consistent with the previous research in Kazakh, Han and Uygur in Xinjiang, which reported a prevalence of diabetes of 3.6%, a mean BMI of 26.6 kg/m^2^ and a mean waist circumference of 88.3 cm in Kazakh [7].

The prevalence of diabetes and obesity varies widely among different ethnicities. A national cross-sectional study in mainland China from 2015 to 2017 reported that the Han ethnicity had the highest prevalence of diabetes (8.8%), and the Tibetan ethnicity had the lowest prevalence (1.9%) among the five investigated ethnicities using the criteria of self-reported diabetes or fasting plasma glucose ≥ 7 mmol/L for diagnosing diabetes, as in our study [10]. A study in the U.S. reported that the diabetic prevalence of Hispanic, black non-Hispanic, Asian non-Hispanic, and white non-Hispanic United States adults was 22.1%, 20.4%, 19.1%, and 12.1% respectively [11]. The prevalence of obesity in Hispanic, black non-Hispanic, Asian non-Hispanic, and white non-Hispanic Americans was 44.8%, 49.6%, 34.2% and 42.2% [12]. We compared the standardized prevalence of diabetes, BMI and waist circumference of our findings with published studies using the same diagnostic criteria of diabetes as in our study in Table 5. Our results indicated that the Kazakh population had a significantly lower prevalence of diabetes but a higher BMI and waist circumference compared with the Han ethnic group in Xinjiang and the overall Chinese population.

**Table 5:** Main characteristics of published studies in comparison to our study

Characteristics	Altay Kazakh	Xinjiang [7]		Overall Chinese [5]	Americans [13]		
		Kazakh	Han		Black	White	Mexican
Number(n)	118,505	3,919	5,583	75,880	4830	9439	3519
Year of survey	2018	2007–2010		2015–2017	2009–2018		
Male(%)	50.7	48.3	48.3	48.5	NA	NA	NA
Age(years)	43.7 ± 14.1	48.7 ± 11.7	52.6 ± 12.7	42.8 ± 0.9	NA	NA	NA
BMI(kg/m^2^)	26.3 ± 4.4	26.6 ± 4.8	25.1 ± 3.5	24.0 ± 0.1	33.5 ± 0.6	33.2 ± 0.6	32.5 ± 0.6
Waist circumference(cm)	87.7 ± 12.7	88.3 ± 13.3	86.8 ± 10.3	83.2 ± 0.8	NA	NA	NA
Standardized Prevalence of diabetes(%)	5.4	3.6	8.1	8.7	14.6	10.6	13.5

The studies all used the same diagnostic criteria of diabetes (fasting plasma glucose ≥ 7.0 mmol/L [126 mg/dL] or diagnosed diabetes). The standardized prevalence of diabetes of published studies were compared with that of the Kazakh population in our study. Abbreviation: BMI, body mass index

In the following multivariate logistic-regression analysis, we found that the most important risk factors for diabetes in the total Kazakh population were hypertension (OR = 3.821), hypertriglyceridemia (OR = 2.757) and hyper-LDL cholesterolmia (OR = 2.331) rather than obesity (OR = 1.453), overweight (OR = 1.265) or central obesity (OR = 1.222). The findings were inconsistent with the previous studies. In general, obesity and central obesity are known to be the greatest risk factors for type 2 diabetes. In the national cross-sectional study in China, family history of diabetes (OR 3.06), obesity (OR 2.62), age (per 10-year increment, OR 2.20) and central obesity (OR 1.49) were most significantly associated with increased risk of diabetes [10]. Family history of diabetes, central obesity, hypertension, and generalized obesity were the most important risk factors for diabetes in India [14]. The general obesity indicator (BMI) and central obesity indicator (waist circumference or waist-to-hip ratio) are independently associated with the risk of type 2 diabetes. The relative risk (RR) for incident diabetes per standard deviation of BMI (RR 1.87) was comparable to that of waist circumference (RR 1.87) or waist-to-hip ratio (RR 1.88) according to a meta-analysis of studies from different geographical areas, including the United States, Europe, and Asia [15]. A recent large global meta-analysis showed that the risk of type 2 diabetes increased by 61% for every 10 cm increase in waist circumference (RR 1.61) and by 72% for every 5 units increase in BMI (RR 1.72) [16]. Ethnic disparity of obesity in the risk of type 2 diabetes has been discussed in genetic studies. In Asian populations, type 2 diabetes begins to manifest at a much lower BMI than in European populations. Asians were shown to have a 15% higher risk of developing diabetes for every unit higher BMI, compared to an 11% higher risk for non-Hispanic whites [17]. In our study, Kazakhs had a 7.6% higher risk of developing diabetes for every unit higher BMI. BMI and waist circumference do not discriminate well between skeletal muscle and body fat. Adipose tissue (AT) is divided into visceral AT and subcutaneous AT. Association of visceral AT accumulation with type 2 diabetes has been confirmed by abundant studies. AT distribution and its impact on glucose metabolism in this population deserves further study.

Notably, the prevalence of diabetes was 14.1% in hypertensive Kazakhs and 11.1% in Kazakhs with dyslipidemia. The multivariate logistic-regression analysis revealed that diabetes was mostly positively associated with hypertension, hypertriglyceridemia and hyper-LDL cholesterolmia. Meanwhile, newly diagnosed diabetes made up 65.5% of the total population with diabetes in our study. More attention should be paid to early screening of diabetes in Kazakhs with hypertension or dyslipidemia.

### Strengths and limitations

There is insufficient data on the prevalence of diabetes and obesity among China’s minority populations. We performed a large epidemiological investigation of the Kazakh in Altay with standardized data-collection protocols with quality control. The large sample size ensured that the metabolic characteristics of the Kazakh ethnic group were analyzed exactly. However, there are several limitations of the study. First, in our epidemiological investigation, 41.17% of the participants were excluded after data cleaning and checking. However, there were 118,505 included participants. We analyzed the demographic characteristics (age and residence) of the included data and found no difference compared to the total. Second, we define diabetes by FPG ≥ 7.0 mmol/L in participants without a prior diagnosis of diabetes. Patients with 2-h plasma glucose ≥ 11.1 mmol/L during OGTT are missed. To minimize the impact of the limitation to the conclusions, a comparison with published studies using the criteria of self-reported diabetes or FPG ≥ 7 mmol/L for diagnosing diabetes as in our study are shown in Table 5. Third, we used BMI and waist circumference, to gauge body composition, but BMI and waist circumference do not discriminate well between skeletal muscle and body fat.

## Conclusion

The Kazakhs in Xinjiang had a considerably low prevalence of diabetes but were more likely to be overweight/obese. Obesity was not the most important risk factor for diabetes in Kazakhs. The rate of diabetes awareness was low. When screening for diabetes in Kazakhs, those with hypertension or dyslipidemia should receive more attention.

### Electronic supplementary material

Below is the link to the electronic supplementary material.

Supplementary Material 1

## Data Availability

The data generated during the current study are available from the corresponding author Mingchen Zhang to zhangmc1015@163.com with publication after approval of a proposal.

## Abbreviations

FPG: Fasting plasma glucose
IFG: Impaired fasting glucose
BMI: Body mass index
TG: Triglycerides
TC: Total cholesterol
LDL: Low-density lipoprotein
HDL: High-density lipoprotein
SBP: Systolic blood pressure
DBP: Diastolic blood pressure
OR: Odds ratio
RR: Relative risk
AT: Adipose tissue
