# Supplementary material for: Kazakh adults in Xinjiang have a prevalent obesity problem but a low prevalence of diabetes
Source: BMC Public Health. 2024 Mar 4;24:689. doi: 10.1186/s12889-024-18228-z (PMC10913663; doi:10.1186/s12889-024-18228-z)
Supplement: Supplementary file 1 — Supplementary Material 1 [file 12889_2024_18228_MOESM1_ESM.docx]

Kazakh adults in Xinjiang have a prevalent obesity problem but a low prevalence of diabetes

Ruiting Shen, Sheng Jiang, Ruirui Cheng, Jinhui Zhuge, Xiaoxiao Li, Hua Yao, Mingchen Zhang*

*** Correspondence:** Mingchen Zhang: [zhangmc1015@163.com](mailto:zhangmc1015@163.com)

Supplementary Material

| Appendix Table 1 | Multivariate adjusted odds ratios (95% CI) for factors predictive of diabetes |
| --- | --- |
| Appendix Table 2 | Odds ratios (95% CI) for factors predictive of diabetes in male and female groups |
| Appendix Table 3 | Odds ratios (95% CI) for factors predictive of diabetes in normal weight, overweight and obesity groups |

**Appendix Table 1. Multivariate adjusted odds ratios (95% CI) for factors predictive of diabetes and IFG**

| Variables | Diabetes | | IFG | |
| --- | --- | --- | --- | --- |
|  | OR（95%CI） | *P* | OR（95%CI） | *P* |
| Overweight | 1.263（1.172-1.362） | ＜0.001 | 1.336（1.292-1.383） | ＜0.001 |
| Obesity | 1.402（1.297-1.515） | ＜0.001 | 1.613（1.552-1.677） | ＜0.001 |
| Central obesity | 1.306（1.230-1.388） | ＜0.001 | 1.176（1.149-1.206） | ＜0.001 |
| Hypertension | 6.305（5.946-6.686） | ＜0.001 | 1.437（1.398-1.478） | ＜0.001 |
| Hypercholesterolemia | 1.177（1.104-1.256） | ＜0.001 | 1.264（1.215-1.315） | ＜0.001 |
| Hypertriglyceridaemia | 2.522（2.364-2.691） | ＜0.001 | 1.208（1.155-1.264） | ＜0.001 |
| Hyper-LDL-cholesterolemia | 2.405（2.230-2.592） | ＜0.001 | 0.982（0.928-1.039） | 0.532 |
| Hypo-HDL cholesterolemia | 1.157（1.059-1.265） | ＜0.001 | 1.105（1.053-1.159） | ＜0.001 |

Odds ratios adjusted for age, sex and location. Abbreviation: IFG, impaired fasting glucose; LDL, low-density lipoprotein; HDL, high-density lipoprotein.

**Appendix Table 2. Odds ratios (95% CI) for factors predictive of diabetes in male and female groups**

| Variables | Male | | Female | |
| --- | --- | --- | --- | --- |
|  | OR（95%CI） | *P* | OR（95%CI） | *P* |
| Age | 1.039 (1.036-1.041) | ＜0.001 | 1.038 (1.035-1.041) | ＜0.001 |
| Rural (vs. urban) | 0.941 (0.870-1.018) | 0.131 | 1.033 (0.950-1.124) | 0.447 |
| Overweight | 1.358 (1.220-1.510) | ＜0.001 | 1.175 (1.053-1.310) | 0.004 |
| Obesity | 1.687 (1.509-1.886) | ＜0.001 | 1.249 (1.120-1.393) | ＜0.001 |
| Central obesity | 1.129 (1.039-1.227) | 0.004 | 1.326 (1.212-1.451) | ＜0.001 |
| Hypertension | 3.272 (3.009-3.558) | ＜0.001 | 4.670 (4.232-5.154) | ＜0.001 |
| Hypercholesterolemia | 1.159 (1.061-1.266) | ＜0.001 | 1.010 (0.918-1.111) | 0.839 |
| Hypertriglyceridaemia | 2.613 (2.399-2.846) | ＜0.001 | 2.973 (2.677-3.302) | ＜0.001 |
| hyper-LDL-cholesterolemia | 2.016 (1.818-2.236) | ＜0.001 | 2.767 (2.474-3.095) | ＜0.001 |
| Hypo-HDL cholesterolemia | 1.231 (1.100-1.379) | ＜0.001 | 1.176 (1.013-1.366) | 0.033 |

Abbreviation: LDL, low-density lipoprotein; HDL, high-density lipoprotein.

**Appendix** **Table 3. Odds ratios (95% CI) for factors predictive of diabetes in normal weight, overweight and obesity groups**

| Variables | Normal weight | | Overweight | | Obesity | |
| --- | --- | --- | --- | --- | --- | --- |
|  | OR（95%CI） | *P* | OR（95%CI） | *P* | OR（95%CI） | *P* |
| Females sex (vs. males) | 1.084（0.960-1.224） | 0.193 | 1.046（0.956-1.144） | 0.329 | 0.935（0.869-1.006） | 0.073 |
| Age | 1.041（1.037-1.045） | ＜0.001 | 1.035（1.032-1.039） | ＜0.001 | 1.037（1.034-1.040） | ＜0.001 |
| Rural (vs. urban) | 0.981（0.852-1.130） | 0.791 | 1.033（0.931-1.145） | 0.542 | 0.979（0.904-1.061） | 0.609 |
| waist circumference | 1.012（1.005-1.018） | ＜0.001 | 1.017（1.013-1.021） | ＜0.001 | 1.013（1.010-1.016） | ＜0.001 |
| Hypertension | 4.916（4.241-5.697） | ＜0.001 | 4.244（3.801-4.740） | ＜0.001 | 3.021（2.760-3.308） | ＜0.001 |
| Hypercholesterolemia | 1.218（1.032-1.436） | 0.019 | 1.065（0.950-1.195） | 0.282 | 1.049（0.960-1.147） | 0.294 |
| Hypertriglyceridaemia | 3.352（2.802-4.010） | ＜0.001 | 3.029（2.688-3.413） | ＜0.001 | 2.414（2.210-2.637） | ＜0.001 |
| hyper-LDL-cholesterolemia | 3.614（2.983-4.379） | ＜0.001 | 2.691（2.358-3.072） | ＜0.001 | 1.961（1.764-2.179） | ＜0.001 |
| hypo-HDL-cholesterolemia | 1.295（1.026-1.636） | 0.003 | 1.160（0.980-1.373） | 0.085 | 1.199（1.063-1.351） | 0.003 |

Abbreviation: LDL, low-density lipoprotein; HDL, high-density lipoprotein
